# Reduced expression of microRNA-206 regulates cell proliferation via cyclinD2 in gliomas

**DOI:** 10.3892/mmr.2015.3171

**Published:** 2015-01-09

**Authors:** XUE YANG, CHUANBAO ZHANG, TIANZHU GUO, YING FENG, QINGYANG LIU, YAN CHEN, QUANGENG ZHANG

**Affiliations:** 1 Department of Immunology, School of Basic Medical Sciences, Capital Medical University, Beijing 100069, P.R. China; 2 Beijing Neurosurgical Institute, Beijing 100050, P.R. China; 3 Department of Neurosurgery, Beijing Tiantan Hospital, Capital Medical University, Beijing 100050, P.R. China; 4 Beijing Key Laboratory of Metabolic Disturbance Related Cardiovascular Disease, Beijing 100069, P.R. China

**Keywords:** glioma, miR-206, cyclin D2, carcinogenesis

## Abstract

MicroRNAs are short single-stranded non-coding RNA molecules that function as regulators of tumor progression, including regulation of glioblastoma multiforme, which is a World Health Organization grade IV glioma. Based on the results of a microRNA microarray, which included 198 patients with glioma from the Chinese Glioma Genome Atlas data set, it was observed that microRNA-206 (miR-206) was downregulated in high-grade (grades III and IV) gliomas compared with grade II gliomas. In addition, high expression of miR-206 was associated with longer overall survival time in glioma patients. The present study aimed to investigate the biological functions of miR-206 in glioma progression *in vitro* using the LN229 glioma cell line. Cell proliferation was observed to be inhibited subsequent to transfection with miR-206. It was suggested that miR-206 induced cell cycle G_1_/S phase arrest by suppressing the expression of cyclinD2. The results of the present study concluded that miR-206 inhibits glioma progression via the regulation of cyclinD2 and that miR-206 may be a novel biomarker with potential for use as a therapeutic target in gliomas.

## Introduction

Glioblastoma multiforme (GBM) is the most prevalent lethal intracranial tumor in adults (1). It is characterized by extensive intracranial invasion and the patients have been reported to tolerate conventional and advanced treatments during therapy (2). Despite the improved strategies for diagnosis and the aggressive tumor treatments used, the median survival of patients with GBM remains to be approximately one year (3,4). Therefore, more effective targeted therapies are crucial for improving the prognosis of patients with GBM.

MicroRNAs are small non-coding RNA molecules that are comprised of 16~22 nucleotides and downregulate translation by targeting mRNAs (5). These microRNAs bind with the 3′ untranslated regions (3′UTRs) to block complementary sites on their mRNA targets and therefore serve important inhibitory functions in the post-transcription of gene expression, in a similar capacity to that of RNAi (6). Previous studies have indicated that this novel class of gene regulators has an involvement in human cancer progression and tumorigenesis (7).

Previous studies reported that microRNA-206 (miR-206) expression was markedly reduced in osteosarcoma and lung cancer, and that it was necessary for cell growth, migration, apoptosis and invasion (8,9). In human breast cancer and rhabdomyosarcoma, downregulated miR-206 was associated with cell proliferation, migration and metastasis (10–13).

However, the function of miR-206 in gliomas remains to be fully elucidated. In the present study, the aim was to investigate the functional role of miR-206 in gliomas and to further elucidate the mechanism of miR-206 in tumorigenesis and progression. The present study hypothesized that miR-206 acts as a tumor suppressor and suppresses glioma cell proliferation via cyclinD2.

## Materials and methods

### Patients and tissue collection

All patient information was obtained from the Chinese Glioma Genome Atlas (CGGA; www.cgga.org.cn/). The microRNA microarray analysis was conducted on 198 patients with glioma and based on the gene expression microarray and cyclinD2 expression was analyzed in 225 patients with grade II glioma or high-grade gliomas (HGGs), of which 158 patients also underwent microRNA microarray analysis. In the present study, the patients underwent a resection operation between January 2006 and December 2010 and subsequently received adjuvant treatment of temozolomide combined with radiotherapy. The present study was approved by the Beijing Tiantan Hospital (Beijing, China) institutional review board and written informed consent was obtained from all patients.

### Cell lines and cell transfection

The human LN229 GBM cell line was purchased from the Chinese Academy of Sciences Cell Bank (Kunming, China). LN229 cells were cultured in Dulbecco’s modified Eagle’s medium supplemented with 10% fetal bovine serum (FBS), 1% penicillin/streptomycin and 1% glutamine (all Hyclone, GE Healthcare, Little Chalfont, UK). All cells were incubated at 37°C in an atmosphere supplemented with 5% CO_2_ and passaged every 2–3 days. The *Homo sapiens* (hsa)-miR-206 mimics were synthesized by Guangzhou RiboBio Co., Ltd. (Guangzhou, China). The following primer sequence was used in the present study: UGGAAUGUAAGGAAGUGUGUGG. Cells were cultured at a density of 8×10^4^ cells/well in six-well plates. miR-NC and hsa-miR-206 oligosaccharides were transfected into LN229 cells at a final concentration of 50 nmol/l using riboFECTTM CP reagent (Guangzhou RiboBio Co., Ltd.) according to the manufacturer’s instructions. The miR-NC contained a scrambled sequence for the control.

### Luciferase reporter assay

For the luciferase assays, the luciferase reporter vector cyclinD2-3′UTR and the negative control were specifically synthesized by Shanghai GenePharma Co., Ltd. (Shanghai, China). The cells were cultured in 12-well plates and transfected with hsa-miR-206 and co-transfected with the luciferase reporter vector. Following incubation for 48 h, cells were harvested and analyzed for *Renilla* luciferase activity using the Dual Luciferase Reporter Assay System (Promega Corp., Madison, WI, USA) in accordance with the manufacturer’s instructions. *Renilla* luciferase was used for normalization.

### Cell proliferation assays

Cell proliferation was assessed using MTT (Sigma-Aldrich, St. Louis, MO, USA) and colony formation assays. Subsequent to transfection with miR-NC and hsa-miR-206 oligosaccharides for 24 h, LN229 cells were transplanted at a density of 2,000 cells/well with five replicated wells for each group in the 96-well plates. In order to determine relative cell growth, MTT assays were conducted for five consecutive days by adding 20 μl MTT solution (5 mg/ml) to each well and incubating at 37°C for 4 h. To solubilize formazan crystals, 150 μl DMSO (Sigma-Aldrich) was added and the absorbance values were detected at 490 nm using a microplate reader (GloMax^®^-Multi Detection system; Promega Corp.), which were proportional to the number of live cells.

Colony formation assays were performed with 200 cells/group plated in six-well plates, which were transfected for 24 h. Following 10 days of incubation, each well was washed with phosphate-buffered saline (Hyclone, GE Healthcare) and stained with crystal violet (Sigma-Aldrich). All colonies were manually counted by using a microscope (Leica DM6000 B; Leica Microsystems GmbH, Wetzlar, Germany).

### Western blot analysis

Western blot analysis was conducted to assess cyclinD2 expression in transfected cells. The total proteins were isolated from the LN229 cell lines transfected with negative control plasmids and miR-206 mimics for 48 h. These cells were isolated with trypsin-EDTA (Hyclone, GE Healthcare), and lysed in lysis buffer (1% Lgepal CA-630, 150 mM NaCl, 50 mM Tris; Sigma-Aldrich) for a minimum of two hours on ice. Subsequent to quantification of protein, an equal amount of protein (10 μg) was added into the sample wells, separated using 10% SDS-PAGE (Sigma-Aldrich) and transferred to polyvinylidene difluoride membranes (EMD Millipore, Billerica, MA, USA). Western blotting was conducted with monoclonal rabbit anti-cylinD2 immunoglobulin G (IgG) as the primary antibody (1:400; Wuhan Boster Biological Technology, Ltd., Wuhan, China) incubated at 4°C overnight. Monoclonal rabbit anti-GAPDH IgG antibody, incubated at a dilution of 1:1,000 at 4°C overnight (Cell Signaling Technology, Inc., Danvers, MA, USA) was used to ensure equal protein loading. The secondary antibodies were horseradish peroxidase-conjugated anti-rabbit IgG, diluted at 1:5,000 and purchased from Origene (Beijing, China)

### Cell cycle analysis

LN229 cells (1–2×10^4^) treated with negative control and miR-206 mimics were plated in six-well plates. Subsequent to incubation for 48 h, the cells were collected by trypsinization, fixed for 24 h in 70% ethanol (Beijing Modern Oriental Fine Chemistry Co., Ltd, Beijing, China) and stained with propidium iodide Beyotime Institute of Biotechnology, Haimen, China) for 30 min in the dark in a water bath at 37°C according to the manufacturer’s instructions (Beyotime Institute of Biotechnology). The cells were then collected and underwent cell cycle analysis using a flow cytometer (Cytomics FC 500; Beckman Coulter, Inc., Brea, CA, US).

### Statistical analysis

The overall survival time was counted from the date of diagnosis with glioma to mortality or the last follow-up visit. Kaplan-Meier survival curves were analyzed and the overall survival times were assessed using the log-rank test. Student’s t-test (two-tailed) was used to estimate significant differences between groups. P<0.05 was considered to indicate a statistically significant difference. All experiments were performed a minimum of three times and data were analyzed using GraphPad Prism, version 5 (GraphPad Software Inc., La Jolla, CA, USA) and SPSS, version 13.0 (SPSS, Inc., Chicago, IL, USA).

## Results

### miR-206 is downregulated in GBM and associated with poor prognosis in patients with glioma

**Figure 1:**
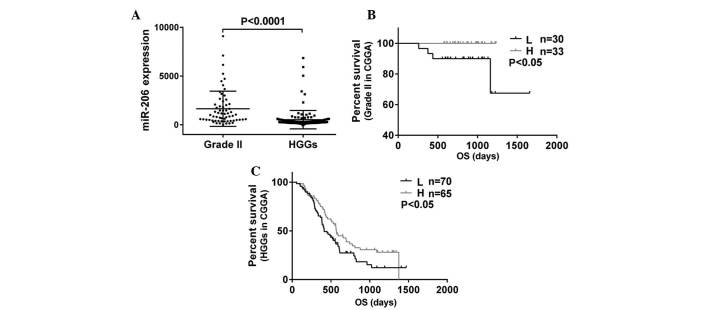
Suppression of miR-206 is associated with high glioma grade and poor prognosis. (A) Expression of miR-206 was significantly lower in HGGs than in grade II glioma (^***^P<0.0001). (B and C) Patients with high expression of miR-206 had a shorter overall survival time than those with a low level of miR-206 in grade II gliomas (^*^P<0.05) and HGGs (^*^P<0.05). miR-206, microRNA-206; HGGs, high grade gliomas; CGGA, Chinese Glioma Genome Atlas; OS, overall survival; L/H, low/high expression of miR-206.

To investigate the tumorigenesis-associated molecular alterations in glioma, the microRNA expression levels were analyzed in 63 patients with grade II glioma and 135 patients with HGGs by microarray analyses. Among these microRNAs, miR-206 expression was observed to be downregulated as the degree of malignancy in gliomas increased (P<0.0001; Fig. 1A). The overall survival time was assessed using Kaplan-Meier survival curve analysis and all survival information used was from the CGGA. The results demonstrated that patients with glioma grade II or HGGs with high expression of miR-206 had a markedly increased rate of progression-free survival as compared with those with low miR-206 expression (P<0.05, P<0.05; Fig. 1B and C). The overall survival curves together with the the miR-206 expression levels in the 198 patients demonstrated that reduced expression levels of miR-206 were associated with poor prognosis in patients with glioma.

### CyclinD2 is a direct target of miR-206

**Figure 2:**
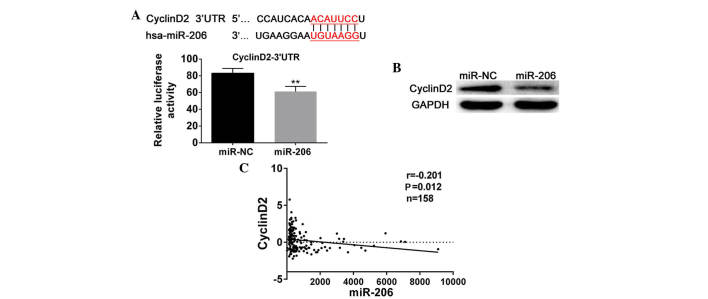
miR-206 directly targets cyclinD2 by binding to the 3′UTR. (A) The binding site of cyclinD2 3′UTR was identified and the underlined base pairs indicate the target region. Luciferase activity was detected by the luciferase reporter assay and normalized to Renilla luciferase activity (^**^P<0.01). (B) Levels of cyclinD2 and GAPDH were detected by western blot analysis. Overexpression of miR-206 significantly reduced the levels of cyclinD2. GAPDH was used as the control. (C) A total of 158 specimens from patients with glioma were detected by microRNA microarray and gene expression microarray and were included to analyze the association between miR-206 and cyclinD2 expression in glioma. The results demonstrated an inverse correlation between the expression of cyclinD2 and miR-206 (Spearman’s rank: r=−0.201, ^*^P=0.012, n=158). miR-206, microRNA-206; UTR, untranslated region; NC, negative control; hsa, *Homo sapiens*.

Based on the above analysis, the possible targets of miR-206 were searched with TargetScan (http://www.targetscan.org/), leading to the identification of cyclinD2. CyclinD2 was observed to share seven imperfect complementary sites with miR-206 and was identified to be important in the cell cycle; therefore, a luciferase reporter assay was designed to verify this. Subsequent to co-transfection with the miR-206 mimics and cyclinD2-3′UTR-plasmids, relative luciferase activity was observed to be significantly reduced (P<0.01; Fig. 2A). The luciferase reporter experiment suggested that cyclinD2 may be a potential target of miR-206. Western blot analysis was also conducted in order to confirm the role of cyclinD2. The results confirmed that miR-206 mimics-transfected cells exhibited reduced cylinD2 expression corresponding to that in the negative control cells (Fig. 2B). Finally, correlation analysis was conducted to investigate the association between the expression of miR-206 and cyclinD2 in 158 patients, and the results demonstrated that there was an inverse correlation between miR-206 and cyclinD2 in gliomas (r=−0.201, P=0.012; Fig. 2C). Based on these results, cyclinD2 was suggested to be a direct target of miR-206 in gliomas.

### CyclinD2 is increased in HGGs and is correlated with poor prognosis

**Figure 3:**
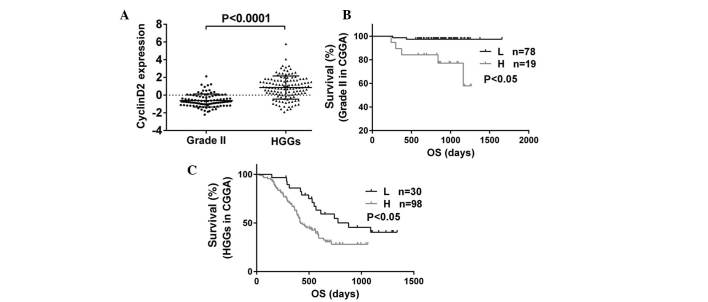
CyclinD2 is increased in HGGs and is correlated with poor prognosis. (A) Levels of cyclinD2 were significantly higher in HGGs than in glioma grade II (^***^P<0.0001, n=225). (B and C) Increased expression of cyclinD2 was correlated with shorter overall survival time either in glioma grade II (^*^P<0.05, n=97) or HGGs (^*^P<0.05, n=128). HGGs, high grade gliomas; CGGA; Chinese Glioma Gene Atlas; OS, overall survival; L/H, low/high expression of CyclinD2.

According to the gene expression microarray, it was observed that with a higher degree of malignancy, cyclinD2 expression was significantly increased (P<0.0001; Fig. 3A). In addition, the correlation between overall survival time and the expression of cyclinD2 was analyzed in 225 patients. The results demonstrated that, independent of the glioma grade, glioma patients with low levels of cyclinD2 expression exhibited a significantly greater survival time, while the survival time of patients with high levels of cyclinD2 was lower (P<0.05, P<0.05; Fig. 3B and C).

### miR-206 inhibits cell proliferation and arrests G_1_/S transition in the cell cycle via targeting cyclinD2 in glioma cell lines

**Figure 4:**
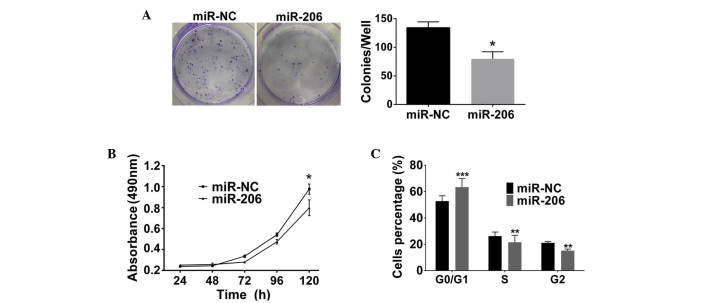
miR-206 inhibits glioma cell proliferation by suppressing the expression of cyclinD2. (A) Colony formation assays were conducted to estimate the growth rate of LN229 cells. Subsequent to treatment with miR-206, the number of colonies formed was reduced compared with that in the negative control cells (^*^P<0.05). (B) An MTT assay was conducted to assess the proliferation of LN229 cells and demonstrated that the absorbance was significantly increased following treatment with miR-206 compared with that in negative control cells. (C) Cell cycle analysis was conducted to validate the percentage of cells in different phases. The number of miR-206-transfected cells was significantly increased in G_0_/G_1_ phase (^***^P<0.001), and simultaneously reduced in S and G2 phase (^**^P<0.01). miR-206, microRNA-206; NC, negative control.

To investigate the biological function of miR-206 in the progression of glioma, a series of overexpression assays were conducted in the GBM cell line LN229. A colony formation assay indicated a significant reduction in colony formation in the miR-206-transfected cells compared with that in the negative control cells (P<0.05; Fig. 4A). In addition, the MTT assay demonstrated a significant reduction in cell growth with cells transfected with miR-206 mimics compared with those of negative control cells at 120 h (P<0.05; Fig. 4B). These assays indicated that miR-206 was associated with glioma cell proliferation. Cell cycle assays demonstrated that the miR-206-transfected cells had a significantly increased percentage of cells in the G_0_/G_1_ phase, whereas a significant reduction in cells in the G_2_ and S phases was observed compared with that in negative control cells (P<0.001, P<0.01 and P<0.01, respectively; Fig. 4C). In conclusion, the results suggested that miR-206 may arrest G_1_/S transition in glioma cell lines via targeting cyclinD2.

## Discussion

Previous studies have indicated that microRNAs regulate gene expression and may also function as tumor suppressors or oncogenes (14). By binding to 3′UTRs, microRNAs suppress the expression of their respective target gene prior to translation, similar to the the mechanism of RNAi (6,15). Furthermore, previous studies have demonstrated that these microRNAs are critical in tumorigenesis and are significant targets for the development of clinical treatments (16,17). Previous studies have identified that miR-206 is downregulated in lung cancer, breast cancer and osteosarcoma (8–10). In breast cancer, miR-206 levels were shown to be correlated with cell growth, clinical stage and lymph node metastasis, and affected the overall survival of patients with breast cancer (10). In lung cancer, as a tumor suppressor, miR-206 was associated with tumor cell migration and invasion (9). Similar effects were also observed in osteosarcoma and in miR-206-transfected cells, where a reduction in cell viability, promotion of cell apoptosis and inhibition of cell invasion and migration were identified (11). However, the function of miR-206 in glioma remains to be fully elucidated.

In the present study, miR-206 was observed to be downregulated in glioma based on microRNA microarray analysis. In addition, the overall survival of patients varied significantly depending on the expression levels of miR-206, suggesting that patients with a high expression of miR-206 had an improved prognosis, based on separate statistical analyses in grade II gliomas and HGGs. Therefore, it was hypothesized that miR-206 may be important in tumorigenesis and the progression of glioma.

To further investigate the function of miR-206 in glioma progression, the target-predicting database Targetscan was searched and cyclinD2 was identified as a potential target of miR-206, which is associated with the cell cycle. miR-206 was previously reported to regulate cyclinD2 in rhabdomyosarcoma (11,12), breast cancer (10,13) and gastric cancer (18). To support this association, a luciferase reporter assay was conducted in the present study, which identified cyclinD2 as a target of miR-206 in gliomas. The results of the western blot assay were also in agreement with this, as cyclinD2 expression was found to be negatively correlated with miR-206 expression. CyclinD2 is a member of D-type cyclins and is crucial in the progression of the cell cycle (19). G_1_ cyclins, including cyclinDs and cyclinEs, combined with cyclin-dependent kinases CDK4 and CDK6, have been reported to be activated in the late G_1_ phase and regulate G_1_/S transition (20). In the process of tumor formation, disruption of cell cycle progression from G_1_ to S phase is commonly observed (21). Based on the results of previous studies, the overall survival was analyzed separately in patients with grade II gliomas and HGGS in regard to the expression of cyclinD2. The results demonstrated that in gliomas, low levels of cyclinD2 may be associated with lower glioma grades and longer survival time, and further confirmed that cyclinD2 may act as a positive regulator in tumorigenesis and function as a tumor oncogene in gliomas. However, miR-206 exhibited the opposite effect, indicating that cyclinD2 is inversely correlated with miR-206 and is negatively associated with the prognosis of gliomas. The correlation between the expression levels of these miR-206 and cyclin D2 is therefore likely to be important in the development of gliomas. Thus, in order to investigate the function of miR-206 in cell proliferation, MTT and colony formation assays were conducted and the results demonstrated that increased miR-206 expression inhibited cell proliferation in GBM. Cell cycle analysis was also conducted in order to detect the percentage of cells in different stages of the cell cycle. This analysis demonstrated that miR-206-transfected cells exhibited a significantly increased G_0_/G_1_ population and a reduction in the S phase population as compared with negative control cells. These results further demonstrated that miR-206 acted as a cell cycle inhibitor, as an increase in the levels of miR-206 expression significantly inhibited transition of LN229 cells from G_0_/G_1_ to S phase. In conclusion, cyclinD2 was a direct target of miR-206 and miR-206 regulated the cell cycle by promoting G_1_/S arrest and suppressing cell proliferation via targeting cylinD2 in gliomas.

In conclusion, to the best of our knowledge, the present study was the first to demonstrate that miR-206 suppresses glioma formation and possibly targets the downstream complementary sites of cyclinD2 to inhibit cancer cell proliferation. In addition, the low expression of miR-206 in patients with glioma was demonstrated to be correlated with poor prognosis. Therefore, it was concluded that miR-206 acts as a tumor suppressor in glioma and regulates cell proliferation and cell cycle arrest by targeting cyclinD2. On the basis of observation and data analysis, miR-206 was suggested to be a novel candidate for use as a prognostic marker in patients with glioma and to have potential for use as a therapeutic target in gliomas.
